# In Vitro and In Vivo Inhibitory Effect of Gujin Xiaoliu Tang in Non-Small Cell Lung Cancer

**DOI:** 10.1155/2018/8936108

**Published:** 2018-09-09

**Authors:** Chao Hou, Dai-Han Zhou, Yong-Jian Wu, Xiao-Jun Dai, Qing-Ying Wang, Yin-Qiu Wu, En-Xin Zhang

**Affiliations:** ^1^Department of Oncology, Yangzhou Hospital of Traditional Chinese Medicine affiliated with Nanjing University of Chinese Medicine, Yangzhou 225000, Jiangsu Province, China; ^2^Department of Oncology, The First Affiliated Hospital of Guangzhou University of Chinese Medicine, Guangzhou 510405, Guangdong Province, China; ^3^Department of Nuclear Medicine, Yangzhou Hospital of Traditional Chinese Medicine, Yangzhou 225000, Jiangsu Province, China

## Abstract

Non-small cell lung cancer (NSCLC) is a serious threat to people's health. This study aims to determine the possible effect of Gujin Xiaoliu Tang (GJXLT) on NSCLC, which is an empirical formula from Professor Dai-Han Zhou. In this study, chromatographic fingerprinting of GJXLT and A549 cell model in vitro and in vivo was established. We cultured A549 cells in vitro and found that GJXLT inhibited A549 cell growth and induced apoptosis. Compared with the control group, the expression of p-STAT3 and VEGF proteins in the GJXLT groups was decreased. Similar findings were also observed in vivo. First, GJXLT inhibited the growth of transplanted tumor and did not reduce the weight of the tumor-bearing mice in comparison with that of the control group. Then, the Ki-67 expression of transplanted tumor in the GJXLT groups was decreased. In addition, the apoptosis rate of transplanted tumor in the GJXLT groups was increased. Overall, our data showed that GJXLT inhibited A549 cell proliferation and induced apoptosis in vivo and in vitro. Furthermore, GJXLT inhibited the growth of lung cancer xenograft in nude mice model with no obvious side effects. The anti-tumor effect of GJXLT might also be related to the inhibition of p-STATS and VEGF expression in the JAK2/STAT3 pathway. Our results demonstrated the potential of GJXLT as a novel treatment for NSCLC.

## 1. Introduction

Lung cancer, a common and severe disease of the respiratory system, ranks first in terms of mortality among all cancers [1]. In China, lung cancer accounts for approximately one-sixth of all new cancer cases, and the death rate is 6.102/1000, which accounts for over 20% of the total tumor mortality [2]. Although new therapies are emerging, the five-year survival rate of lung cancer is less than 20% [3]. Non-small cell lung cancer (NSCLC) accounts for approximately 85% of all lung cancer cases [4, 5]. Most NSCLC is found in the middle and late stages, and its five-year survival rate is low. Dual chemotherapy based on platinum is a standard treatment for advanced NSCLC, but the curative effect of chemotherapy in metastatic NSCLC patients is extremely limited, of whom the response rate is less than 35% and the median total survival period is 6.9 to 11.3 months [6, 7]. In addition, these treatments, including chemotherapy and radiotherapy, often have adverse side effects [8, 9]. Therefore, new anti-cancer drugs with fewer side effects are needed.

Chinese herbs have been used to treat malignant tumor for hundreds of years. Modern research shows that they can inhibit tumor growth in various ways, such as inducing cell cycle arrest and attenuating the tumor-associated macrophage-stimulated proliferation [10–12]. Gujin Xiaoliu Tang (GJXLT) is an empirical formula based on the theory of “benefiting vital energy and eliminating phlegm” and has been used in local hospitals for decades. Previous clinical studies have revealed that combined with chemotherapy, GJXLT plays an important role in the treatment of cancer, including enhancing the effect of chemotherapy, relieving the pain of patients, and prolonging patient survival time [13, 14]. However, no study has determined whether GJXLT possesses anti-tumor effects and its possible anti-cancer mechanism. Therefore, the aim of this study was to investigate the effects of GJXLT on NSCLC in vitro and in vivo and clarify its underlying mechanisms.

## 2. Materials and Methods

### 2.1. Preparation of GJXLT

Raw herbs were obtained from the Chinese pharmacy of The First Affiliated Hospital of Guangzhou University of Traditional Chinese Medicine. The eight herbs of GJXLT,* Gecko*,* Semen Coicis*,* Ginseng*,* Stemona*,* Corium Bufonis*,* Arisaema consanguineum*,* Black Nightshade*, and* Fritillaria thunbergii Miq*, were mixed in a ratio of 1:6:3:4:2:3:4:4 and boiled in 500 mL of sterile water for 30 min. The criteria for identifying the quality of the herbs used were in accordance with the 2005 edition of the Chinese Pharmacopoeia (Chinese Pharmacopoeia Commission, Pharmacopoeia of the People's Republic of China, Beijing: People's Medical Publishing House; 2005). Prior to their use in experiments, the herbs were tested for heavy metals, microbial contamination, and residual pesticides; all results met the safety standards in China. Laboratory personnel were blinded to the identity of the herbs. A trained technician prepared the decoction according to a standardized procedure. The aqueous extracts of the raw ingredients of GJXLT were condensed approximately into 4.22 g/mL and stored at -4°C. For in vitro experiments, a quantified amount (50 mL) of the GJXLT extract was processed with a freeze dryer to obtain crystal powder. Freeze-dried powder was dissolved in culture medium and filtered and stored as a stock solution (142 mg/mL) at -20°C [11].

### 2.2. Chemicals and Reagents

3*-*(4,5-DimethylthiazoL-2-yl)-2,5-diphenyltetrazolium bromide(MTT) was purchased from Sigma Chemical Co. (St. Louis, USA). Annexin V-FITC/PI Apoptosis Kit, TUNEL Apoptosis Kit, and Ki-67 Immunohistochemical Monitoring Kit were purchased from Proteintech Group, Inc. (Chicago, USA). Antibodies against *β*-actin, STAT3, p-STAT3, and VEGF were also purchased from Proteintech Group, Inc. (Chicago, USA). Cisplatin (DDP) was purchased from Qilu Pharmaceutical Company (Ji'nan, China).

### 2.3. Cell Culture

Human NSCLC A549 cells were purchased from the Cell Bank of the Chinese Academy of Sciences of Shanghai. Cells were cultured in RPMI1640 medium with 10% fetal bovine serum (FBS, Gemini, USA), 100 U/mL penicillin, and 100 mg/mL streptomycin in a humidified atmosphere with 5% CO_2_ at 37°C (Thermo Fisher Science, MA, USA). The cells with 80% confluences were treated with different concentrations of GJXLT.

### 2.4. Animals

BALB/c female nude mice (4 weeks old, weighing 18–22 g) were maintained under specific pathogen-free conditions with constant temperature (23 ± 2°C) and controlled light (12 h light:12 h dark). The study was approved by the Institutional Animal Care and Use Committee (animal authorization reference number: SCXK2013-0034) at Guangzhou University of Chinese Medicine (Guangdong, China). Animal welfare and experimental procedures were strictly carried out in accordance with the Guide for the Care and Use of Laboratory Animals (The Ministry of Science and Technology of China, 2006). All efforts were made to minimize animals' suffering and to reduce the number of animals used.

### 2.5. Chemical Analysis of GJXLT

We used the Waters High-Performance Liquid Chromatography (HPLC) system to analyze the chemical composition of GJXLT. The system comprised a 626 pump, a 600 s controller, and a 996 photodiode array detector. A C18 column (250 mm × 4.0 mm, 5 *μ*m, ACE, UK) was used as solid phase while acetonitrile (DUKSAN)-H_2_O containing 0.05% KH_2_PO_4_ (pH = 2.5) was utilized as mobile phase. The flow rate was 1 mL/min, and the detection wavelength was 254 nm.

### 2.6. Cell Proliferation Assay

MTT assay was used to measure cell proliferation. Briefly, A549 cell lines were seeded in 96-well culture plates at a density of 5 × 10^3^ cells per well in complete medium, incubated overnight to allow attachment, and divided into different groups (n = 6). The cells in the control group were treated with culture medium, while others were treated with culture medium containing different concentrations of GJXTL (8.88–568.00 *μ*g/mL) for 12, 24, and 36 h. Then, the cells were incubated with 100 *μ*L of 0.5 mg/mL MTT at 37°C for 4 h, and the precipitate was dissolved in 150 *μ*L dimethylsulfoxide (DMSO). After shaking for 10 min, optical density (OD) was measured at a wavelength of 570 nm and a reference wavelength of 0 nm using a multimode reader (Synergy HTX, BioTek, USA). The inhibition rate was calculated as follows: Inhibition rate (%) = [average OD value (control) − average OD value (medication)]/average OD value (control) × 100%. The IC50 value was calculated on the non-linear regression fit method by the SPSS statistics software (Statistical Product and Service Solution, IBM, New York, USA).

### 2.7. Cell Apoptosis Assay

Annexin V-FITC/PI stained fluorescence-activated cell sorter (FACS) and Annexin V-FITC stained fluorescence microscopy were used to measure cell apoptosis. Briefly, A549 cell lines were seeded at a density of 2 × 10^4^ cells/well overnight, divided into different groups (n = 3), and then treated with GJXLT at different concentrations for 24 h. All cells were harvested through trypsinization and washed twice with cold PBS (0.15 mol/L, pH 7.2). The cells were centrifuged at 1000 r/min for 5 min. Then, the supernatant was discarded and the pellet was resuspended in 1× binding buffer at a density of 1.0 × l0^6^ cells/mL. A total of 100 *μ*L of the sample solution was transferred to a 5 mL culture tube and incubated with 5 *μ*L of FITC-conjugated Annexin V and 10 *μ*L of PI for 15 min at room temperature in the dark. A total of 400 *μ*L of 1× binding buffer was added to each sample tube, and the samples were analyzed by FACS (Becton Dickinson, USA) using Cell Quest Research Software (Becton Dickinson, USA).

### 2.8. Western Blot

The cells were treated with GJXTL at different concentrations for 24 h and lysed with RIPA buffer for 30 min on ice. Then, the cells were centrifuged at 12000 rpm for 15 min at 4°C, and the supernatant was collected. The protein concentration was measured by the BCA method. Equal amount of protein (80 *μ*g) from each sample was separated by SDS-PAGE and transferred onto PVDF membranes. The membranes were blocked with 7% skimmed milk for 2 h at room temperature and incubated with different primary antibodies overnight at 4°C. After washing with TBST (10 mM Tris-HCL, pH 7.4, 150 mM NaCl, 0.05% Tween 20) for three times, the membranes were incubated with the appropriate horseradish peroxidase-conjugated secondary antibodies (1:5000 dilutions). Anti-*β*-actin antibody was used as a loading control. The protein bands were detected by employing an enhanced chemiluminescence system. Protein quantitative analysis was conducted by utilizing the Quantity One 4.6.3 software.

### 2.9. Tumor Growth Assays

A549 cells were cultured and collected by centrifugation (1000 rpm, 5 min) and washed twice with ice-cold PBS. Then, 7 × 10^6^ A549 cells in 200 *μ*L saline were injected into the right flank of BALB/c nude mice. Most mice formed tumors one week after injection. Then, mice were randomly divided into five groups (n = 10) according to tumor volumes. Saline, cisplatin (2 mg/kg), and GJXLT of different concentrations (0.5, 1, and 2 g/mL) were administered or injected intraperitoneally to each group daily since day 0. Tumor length and width were measured with a Vernier caliper every four days. Tumor volumes were measured and calculated using the equation volume= a*∗*b^2^/2, where “a” is the maximal width and “b” is the maximal orthogonal width. The inhibition rates of the tumors were calculated as follows: inhibition rate (%) = [average tumor volume (control) − average tumor volume (experimental)]/average tumor volume (control) × 100%. The minimum and maximum values should be excluded for the calculation of average tumor volumes. On the 21st day, mice were anesthetized and the tumors were removed.

### 2.10. Immunohistochemistry

Paraffin-embedded tumor sections (3 *μ*m) were soaked in an antigen retrieval buffer containing 10 mM sodium citrate (pH 6.0) and treated twice with microwave irradiation (650 W) for 10 min. Antibody against Ki-67 was incubated with the tumor sections overnight. After washing with PBS, the tumor sections were incubated with biotinylated secondary antibodies and an avidin/streptavidin-based detection system, followed by treatment with a 3,3′-diaminobenzidine tetrahydrochloride (DAB) staining system (Merck Millipore, Billerica, MA, USA) and counterstaining with hematoxylin. The stained sections were imaged using a microscope (Olympus BX61, Tokyo, Japan). The positive expression rates of Ki-67 were calculated as follows: positive expression rates of Ki-67 (%) = the number of positive expression cells/the total number of cells × 100%.

### 2.11. TUNEL Assay

The apoptotic cells in paraffin-embedded tumor sections were detected with an in situ cell death detection kit based on the labeling of DNA strand breaks. The tumor sections were labeled successively by Streptavidin-FITC and POD-conjugated Anti-FITC, followed by treatment with a DAB staining system (Merck Millipore, Billerica, MA, USA) and counterstaining with hematoxylin. The stained sections were imaged using a microscope (Olympus BX61, Tokyo, Japan).

### 2.12. Determination of Body Weight Changes in Mice

Weight of the mice was measured before the experiment. At the end of the experiment, the weight increase rate of every group was calculated as follows: weight increase rate (%) = [average weight (after the experiment) − average weight (before the experiment)]/average weight (before the experiment) × 100%.

### 2.13. H&E Staining of Hepatic and Nephridial Tissues

After the treatment, all mice were anesthetized, and the livers and kidneys of mice were removed, cut at 5 *μ*m intervals, and stained with H&E. The stained sections were imaged using a microscope (Olympus BX61, Tokyo, Japan).

### 2.14. Blood Cell and Blood Chemistry Measurements

Blood samples were collected from mice under terminal anesthesia through cardiac punctures. Clear blood samples were prepared, and blood cells were measured with a three-classification blood cell analyzer (pocH-100i, SYSMEX, Japan). Clear serum samples were prepared and measured with an automatic clinical biochemistry analyzer (ADVIA 1800, SIEMENS, Germany).

### 2.15. Statistical Analysis

All data are presented as the mean ± SEM (standard error of mean) and obtained from at least three independent experiments. The Mann-Whitney U test was used to determine the significance of between-group differences. Statistical significance was set at* p* < 0.05. All* p *values were two-tailed, and all statistical analyses were performed with the SPSS statistics software (Statistical Product and Service Solution, IBM, New York, USA).

## 3. Results

### 3.1. Chromatographic Fingerprinting of GJXLT

**Figure 1:**
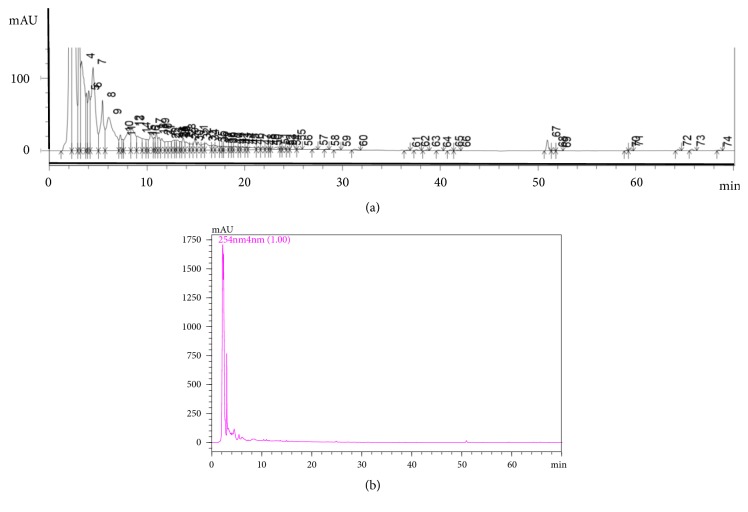
Chromatographic fingerprinting of GJXTL. (a) PDA polychromatic spectrogram of GJXTL. A total of 74 peaks were identified as the characteristic profile of GJXTL extract. (b) Simplified chromatographic fingerprinting of GJXTL.

GJXLT extract was isolated with the HPLC system, and its PDA polychromatic spectrogram was established as shown in Figure 1(a). Figure 1(a) shows the complexity of GJXLT chemical composition. A total of 74 peaks were identified as the characteristic profile of GJXTL extract (Figure 1(a)). The simplified chromatographic fingerprinting of GJXTL was established, as shown in Figure 1(b).

### 3.2. GJXLT Inhibited A549 Cell Growth

**Figure 2:**
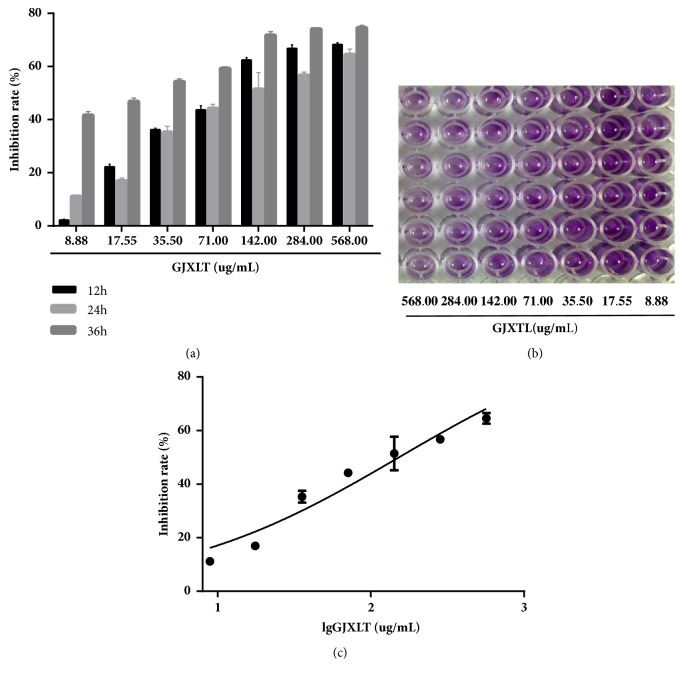
Inhibition rates of different concentrations of GJXTL. (a) Inhibition rates of different concentrations of GJXTL at different time points. (b) Photograph of the 96-well culture plates, in which A549 cells were treated with complete medium containing different concentrations of GJXTL for 24 h, incubated with MTT, and dissolved in DMSO. (c) Scatter plot of inhibitory rate of different concentrations of GJXTL on A549 cells for 24 h. Data are presented as the mean ± SD obtained from at least three independent experiments.

MTT assays were performed with the NSCLC cell line A549 after treatment with different concentrations of GJXLT (8.88–568.00 *μ*g/mL) for 12, 24, and 36 h. Compared with the 0 *μ*g/mL GJXLT group, the average OD values of the 8.88–568.00 *μ*g/mL GJXLT groups were approximately considerably lower at the same time point, which was positively correlated with the concentration. In the same GJXLT group concentration (8.88–568.00 *μ*g/mL), the OD values at 24 and 36 h were approximately considerably lower compared with that at 12 h, which was positively correlated with the time of action within 36 h (Table 1). The inhibition rates of different concentrations of GJXLT (8.88–568.00 *μ*g/mL) at different time points showed that the inhibitory effect of GJXTL on the proliferation of A549 cells was time- (within 36 h) and concentration-dependent (Figure 2(a)). Patients usually take one dose of Chinese herbal medicine every 24 h; therefore, we photographed the 96-well culture plates, in which A549 cells were treated with culture medium containing different concentrations of GJXLT for 24 h, incubated with MTT, and dissolved in DMSO (Figure 2(b)). The half-maximal inhibitory concentration (IC_50_) of GJXLT on A549 cells for 24 h was also calculated. Scatter plots were obtained on the basis of inhibition rate (y) and the numerical value of the concentration of each group (lgx) (Figure 2(c)). According to the scatter diagram, it was inferred that lgx is linear with y. The linear regression equation was obtained by SPSS statistical software: y = 50 was substituted into the equation to obtain the half-maximal inhibitory concentration (IC_50_) = 151.06 ± 13.07 *μ*g/mL.

### 3.3. GJXLT Induced A549 Cell Apoptosis

**Figure 3:**
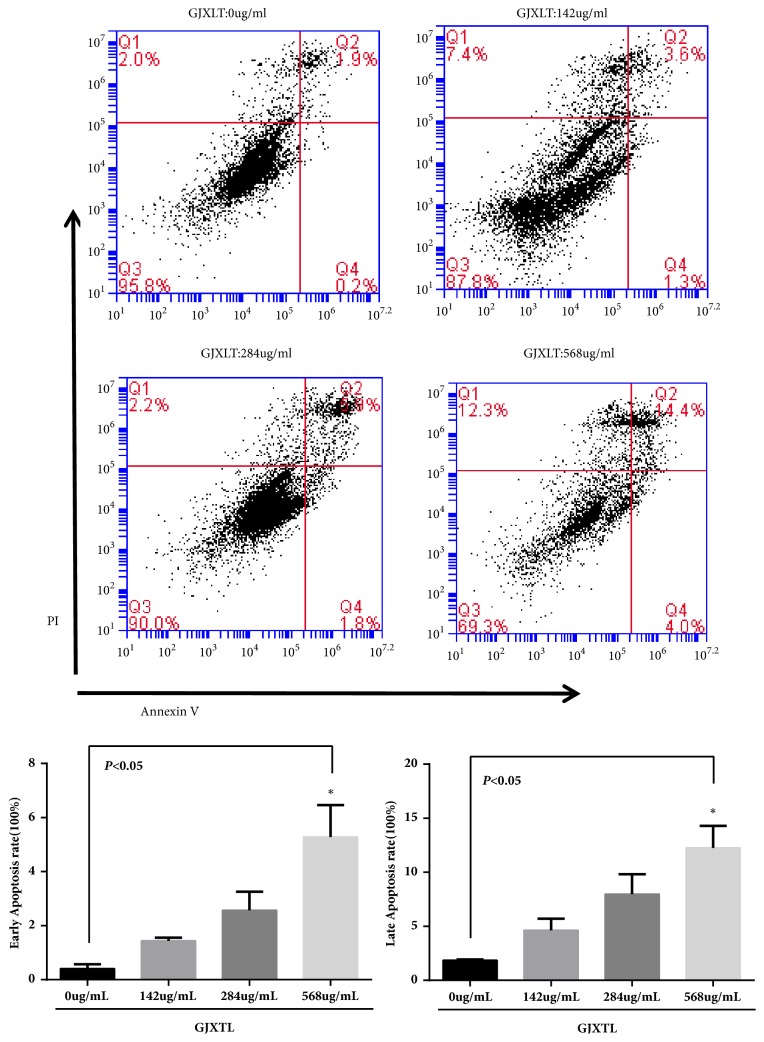
Early and late apoptosis induction of GJXTL against A549 cells for 24 h. Data represent the mean ± SD, n = 3, *∗p *< 0.05 vs. GJXLT group (0 *μ*g/mL).

By staining cells with Annexin V-FITC and PI, FACS was used to distinguish and quantitatively determine the percentage of dead, viable, apoptotic, and necrotic cells after treatment with GJXTL at different concentrations for 24 h (Table 2 and Figure 3). After 24 h, the percentage of early apoptotic and advanced apoptotic cells obviously increased from (0.4066 ± 0.1950)% and (1.8600 ± 0.2821)% in the GJXTL group (0.00 *μ*g/mL) to (5.28 ± 1.31)% and (12.2633 ± 1.9886)% in the GJXTL group (568.00ug/mL), respectively. The percentage of early and advanced apoptotic cells in the GJXTL group (142.00 *μ*g/mL) and the GJXTL group (284.00ug/mL) was also higher than that of the GJXTL group (0.00 *μ*g/mL).

### 3.4. GJXTL Reduces the Expression of Related Proteins in the JAK2/STAT3 Signal Pathway In Vitro

**Figure 4:**
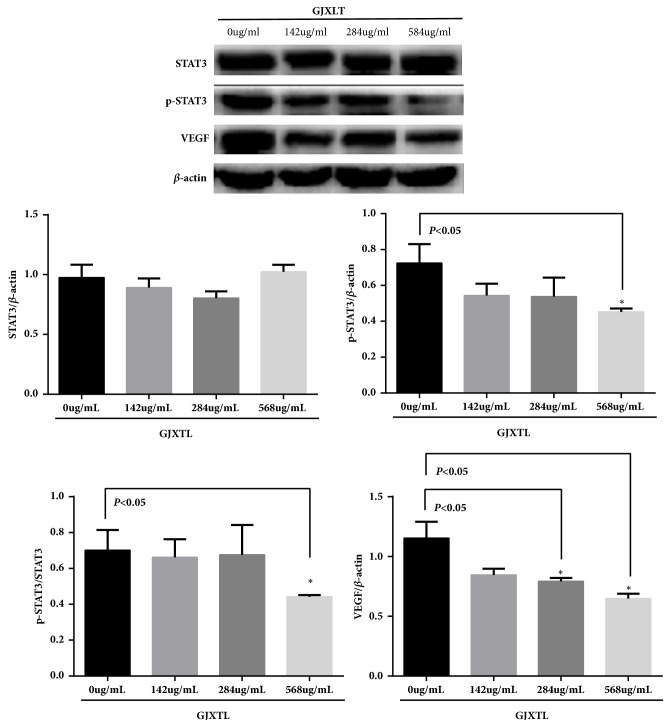
Effects of GJXTL on relative expression of STAT3, p-STAT3, and VEGF protein activity in the JAK2/STAT3 signal pathway. Data represent the mean ± SD, n = 3, *∗p *< 0.05 vs. GJXLT group (0 *μ*g/mL).

To determine whether GJXLT can suppress JAK2/STAT3 pathway activation, Western blot was used to examine STAT3, p-STAT3, and VEGF protein activity changes in the JAK2/STAT3 pathway after treatment with GJXTL at different concentrations for 24 h (Figure 4). Compared with the 0.00 *μ*g/mL GJXTL group, the expression of p-STAT3 protein and p-STAT3/STAT3 ratio in the 568.00 *μ*g/mL GJXLT group were considerably lower, and that in other GJXLT groups were also lower, indicating concentration dependency. Compared with the 0.00 *μ*g/mL GJXTL group, the expression of VEGF protein in the 284.00, and 568.00 *μ*g/mL GJXLT groups was considerably lower, and that in 142.00 *μ*g/mL GJXLT group was also lower, which showed concentration dependency. No significant difference was observed in STAT3 protein expression between each group.

### 3.5. GJXLT Suppresses A549 Tumor Growth in Xenograft Mice

**Figure 5:**
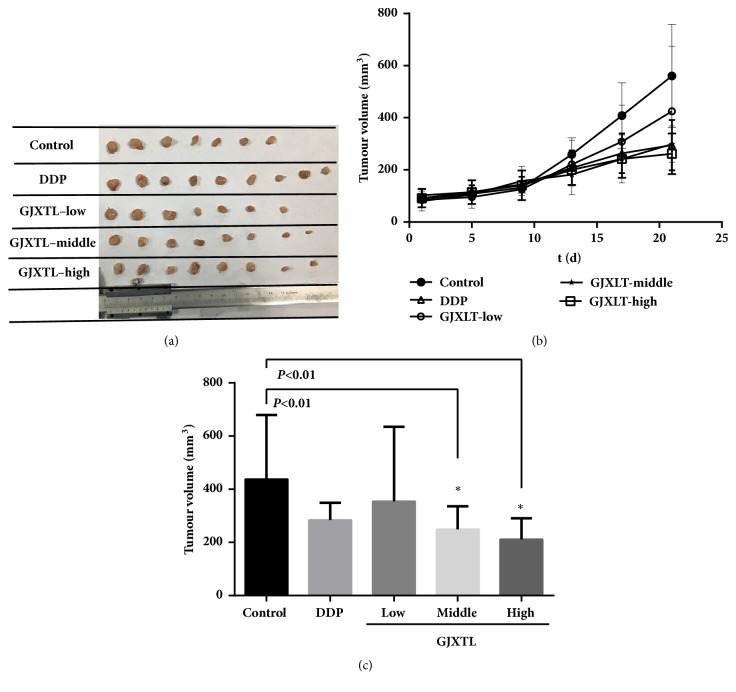
Tumor inhibitory effect of GJXTL in vivo. (a) The tumor was excised from animals after treatment. (b) The tumor volumes were measured once every four days. (c) The comparison of stripped tumor volume of five groups. The minimum and maximum values should be excluded for the calculation of average tumor volumes. Data represent the mean ± SD, *∗p *< 0.01 vs. control.

We evaluated the anticancer effect of GJXLT on female nude mice bearing A549 tumor. After treatment for four weeks, all mice were anesthetized, and the tumors were removed. DDP (2 mg/kg) and middle and high concentration of GJXLT decreased tumor volume to some extent, and a statistical difference was observed in comparison with the control group. Low concentration of GJXLT slightly decreased tumor volume, and no statistical difference was observed in comparison with the control group. In A549 xenograft mice, the tumor volume was decreased by GJXLT dose-dependently (Figure 5).

### 3.6. GJXLT Reduced the Protein Expression of Ki-67

**Figure 6:**
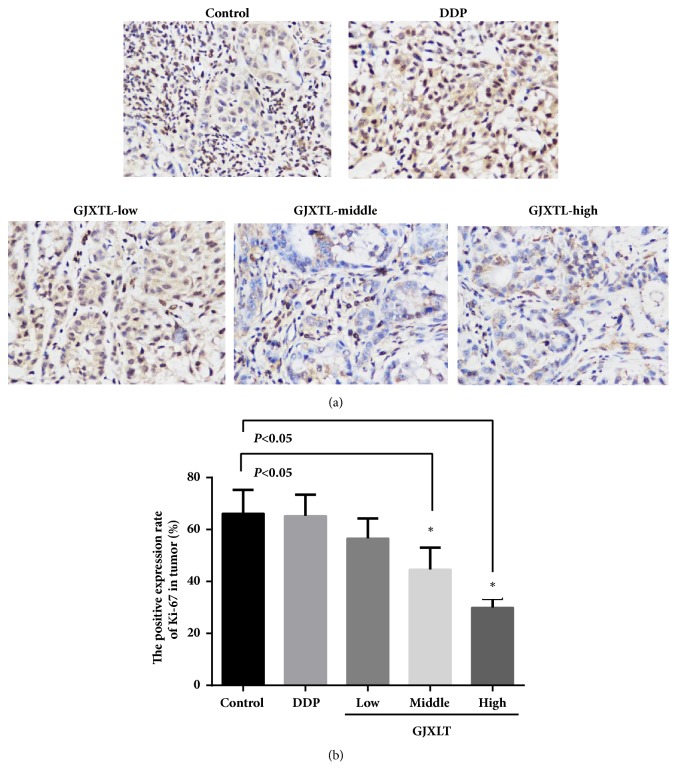
GJXLT reduces Ki-67 protein expression in vivo. (a) Representative IHC staining of Ki-67 (IMC, 400×). (b) Comparison of the positive expression rates of Ki-67 protein of five groups. Data represent the mean ± SD, n = 5, *∗p *< 0.05 vs. control.

GJXLT decreased the protein expression of Ki-67 in A549 tumor tissue in a dose-dependent manner (Table 3 and Figure 6). The positive expression rates of Ki-67 in the middle and high dose groups of GJXLT were significantly lower compared with those of the control group.

### 3.7. GJXLT Induced A549 Cell Apoptosis In Vivo

**Figure 7:**
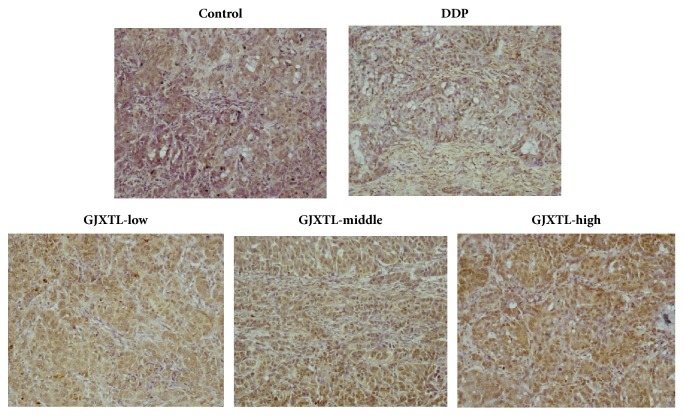
GJXTL induced A549 cell apoptosis in vivo. Compared with the control group, the apoptotic cells in GJXLT groups increased in a dose-dependent manner (200×).

TUNEL assay was used to examine the situation of cell apoptosis in stripped tumor after treatment. The apoptotic cells of A549 in GJXLT groups were increased in a dose-dependent manner (Figure 7).

### 3.8. GJXTL Caused No Significant Side Effects In Vivo

**Figure 8:**
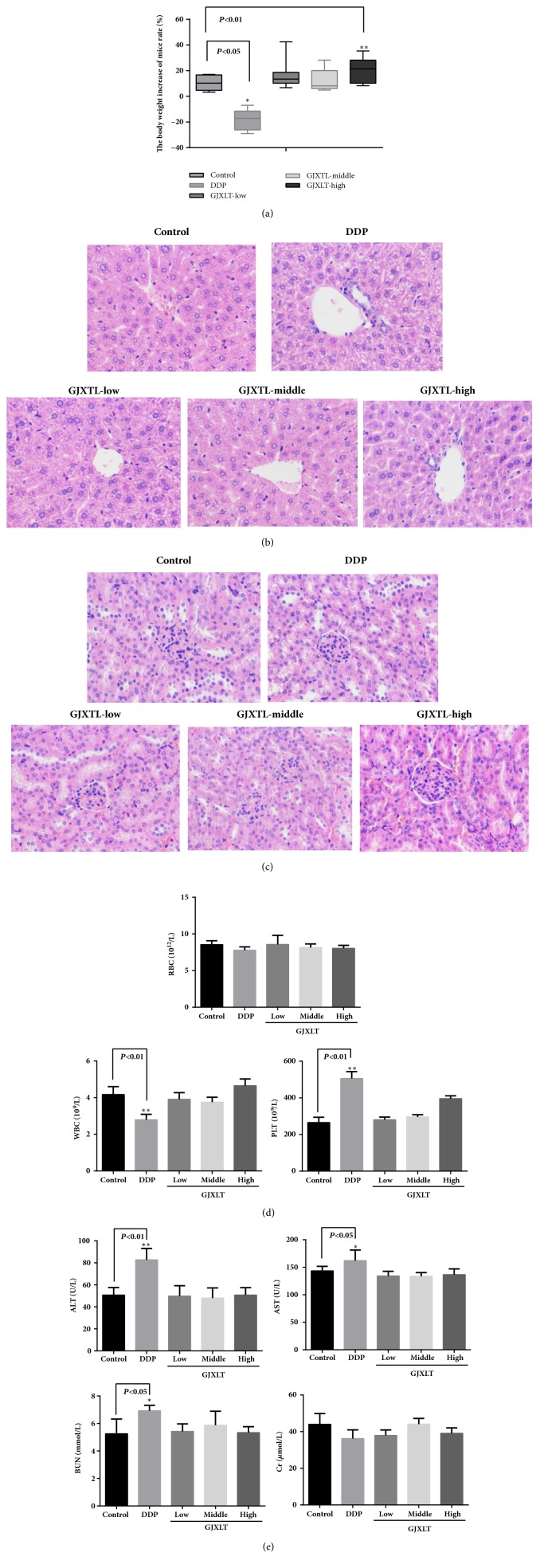
GJXTL caused no significant side effects in vivo. (a) GJXTL increased the body weight of A549 xenograft mice. (b) Representative HE staining of hepatic tissue (HE, 400×). Compared with the control group, the liver cells in the DDP group had mildly balloon-like change and edema, but the liver cells in the GJXTL group had no pathological changes. (c) Representative HE staining of nephridial tissue (HE, 400×). Compared with the control group, the nephridial tissue of the other groups had no pathological changes. (d) GJXTL did not decrease the number of blood cells of A549 xenograft mice. (e) GJXLT caused no change in liver and kidney function in A549 xenograft mice. Data represent the mean ± SD, n = 5, *∗p *< 0.05 and *∗∗p *< 0.01 vs. control.

After treatment for four weeks, GJXTL increased the body weight of A549 xenograft mice dose-dependently. As shown in Figure 8(a), high concentration of GJXTL increased the body weight to some extent, and no statistical difference was observed in comparison to the control group. Low and middle concentrations of GJXTL slightly decreased and increased the body weight, respectively, and no statistical difference was observed in comparison to the control group. In addition, GJXLT caused no change in liver and kidney function in A549 xenograft mice (Figures 8(b), 8(c), 8(d), and 8(e)).

## 4. Discussion

Traditional Chinese medicine (TCM) has potential anticancer effects worthy of study. However, rigorous and systematic investigation is necessary to ensure the efficacy of evidence-based herbal formulas and transform traditional herbal practices into science-based medicines [10–12, 15–17]. Professor Dai-Han Zhou holds that the formation of lung cancer is related to TCM pathogenesis theory of spleen deficiency and phlegm-turbid stagnation. Professor Zhou followed the method of “invigorating qi and removing phlegm” and formulated the GJXLT formula, which is also named Yiqi Huatan formula and has been suggested in the treatment of lung cancer from over 50 years of clinical experience. Clinical reports revealed that GJXLT can prolong the median survival time in NSCLC patients [13, 14]. Pharmacological experimental studies have demonstrated that the effective components of the herbs in GJXLT formula, such as bufalin of Corium Bufonisgecko and ginsenoside of Ginseng, play a vital role in the anti-tumor effect by inhibiting cellular proliferation [18, 19]. In this study, we confirmed the anti-tumor efficacy of GJXLT. GJXLT can inhibit cell growth of human lung cancer A549 (Table 1 and Figure 2). In addition, it can also inhibit the growth of human A549 xenograft tumors (Figure 8).

Fingerprinting of the formula was established to control the quality of GJXLT (Figure 1). However, the main effective ingredients of GJXLT extract still remain to be further identified for the purpose of optimizing the formula and discovering Chinese herbal medicines with potential anti-tumor effects.

Apoptosis is an important regulatory factor in the development process, maintenance of homeostasis, and elimination of damaged cells. It is the result of complex interaction between apoptotic and anti-apoptotic molecules [20–22]. In this study, we found that GJXLT could induce A549 cell apoptosis in vivo and vitro (Table 2, Figures 3 and 7). However, whether the effect of GJXTL is related to the regulation of anti-apoptotic Bcl-2, pro-apoptotic Bax, and other molecules such as Caspase protein family needs to be further investigated.

Tumor angiogenesis is crucial in tumor growth and metastasis. The vascular endothelial growth factor (VEGF) is an important regulatory factor for tumor angiogenesis and has become a target for cancer treatment [23–25]. In this study, we found that the GJXLT formula dose-dependently suppressed the expression of VEGF in vitro (Table 3 and Figure 4). Therefore, VEGF may be one of the anti-tumor targets of GJXTL.

The JAK2/STAT3 signaling pathway is closely related to tumor development. In many cases, TAM-derived IL-6 and other cytokines activate STAT3 to promote tumor development by inducing proliferation and inhibiting apoptosis [26]. Many new drugs, such as DNA methyltransferase inhibitors, can promote tumor cell apoptosis, cell proliferation, angiogenesis, and distant metastasis by inhibiting the phosphorylation of STAT3 [27–30]. In addition, studies have shown that activated STAT3 (p-STAT3) can induce the expression of VEGF [31, 32]. Our study found that GJXLT inhibited the expression of VEGF and p-STAT3 in vitro (Table 3 and Figure 4). Therefore, p-STAT3 may be an effective target for inhibiting angiogenesis by reducing the expression of VEGF in the tumor.

## 5. Conclusion

This study confirmed the anti-tumor effect of GJXLT and preliminarily revealed the anticancer mechanism of GJXLT. GJXLT can inhibit A549 cell proliferation and induce apoptosis in vivo and in vitro. Moreover, it also inhibits the growth of lung cancer xenograft in nude mice model with no obvious side effects. The anti-tumor effect of GJXLT might be related to the inhibition of p-STATS and VEGF expression in the JAK2/STAT3 pathway.

## Figures and Tables

**Table 1:** Cytotoxicity of different concentrations of GJXTL against A549 cells at different time points.

GJXLT (ug/ml)	n	OD value		
		12h	24h	36h
0	6	0.8467±0.0102	1.0446±0.1838	1.4629±0.1468
8.88	6	0.8293±0.1634	0.9285±0.0765	0.8423±0.0826*∗∗*
17.55	6	0.6568±0.0711*∗∗*	0.8687±0.0209*∗*^▲^	0.7755±0.1327*∗∗*^▲^
35.50	6	0.5409±0.0646*∗∗*	0.6798±0.0677*∗∗*	0.6658±0.1141*∗∗*
71.00	6	0.4765±0.0246*∗∗*	0.5897±0.0930*∗∗*^▲^	0.5983±0.0497*∗∗*^▲^
142.00	6	0.3139±0.0205*∗∗*	0.5653±0.1334*∗∗*^▲^	0.4321±0.0327*∗∗*^▲^
284.00	6	0.2788±0.0292*∗∗*	0.4621±0.0081*∗∗*^▲^	0.3836±0.0498*∗∗*^▲^
568.00	6	0.2705±0.0542*∗∗*	0.3648±0.0268*∗∗*^▲^	0.3803±0.0581*∗∗*^▲^

Data represent mean ± SD. *∗p *< 0.05 and *∗∗p *< 0.01 indicated significant differences compared with the 0 *μ*g/mL GJXLT group at the same time point. ^▲^*p *< 0.01 indicated significant differences compared with the OD value at 12 h in the 8.88–568.00*μ*g/mL GJXLT group.

**Table 2:** Apoptotic rate of A549 cells treated with different concentrations of GJXTL for 24 h.

GJXTL (ug/mL)	n	Viable cells (%)	Early apoptotic cells (%)	Advanced apoptotic cells (%)
0.00	3	95.1400±0.6161	0.4066±0.1950	1.8600±0.2821
142.00	3	83.5933±2.9206	1.4467±0.3465	4.6900±0.1931
284.00	3	80.0533±2.8002	2.5667±0.8159	7.9867±1.8336
568.00	3	71.8667±2.2550	5.28±1.31*∗*	12.2633±1.9886*∗*

Data represent the mean ± SD, n = 3, *∗p *< 0.05 vs. GJXLT group (0 *μ*g/mL).

**Table 3:** GJXLT reduces Ki-67 protein expression in vivo.

Group	n	The positive expression rates of Ki-67 (%)
Control	5	66.25±9.06
DDP	5	65.28±8.14
GJXLT-low	5	56.62±7.71
GJXLT-middle	5	44.61±8.39^*∗*^
GJXLT-high	5	29.94±3.41^*∗*^

Data represent the mean ± SD, n = 5, *∗p* < 0.05 vs. control.

## Data Availability

All relevant data are within the paper and its Supporting Information file.

## Supplementary Materials

Supplementary Materials The Supplementary Material includes the raw date of the experiment, in which the blood routine biochemical results of mouse experiment and the data related to the molecular cell animal experiments were provided respectively.
