# Supplementary material for: In Vitro and In Vivo Inhibitory Effect of Gujin Xiaoliu Tang in Non-Small Cell Lung Cancer
Source: Evid Based Complement Alternat Med. 2018 Sep 9;2018:8936108. doi: 10.1155/2018/8936108 (PMC6151250; doi:10.1155/2018/8936108)
Supplement: Supplementary Materials — The Supplementary Material includes the raw date of the experiment, in which the blood routine biochemical results of mouse experiment and the data related to the molecular cell animal experiments were provided respectively. [file 8936108.f1.pdf]

The OD value of different concentrations of GJXTL against A549 cells at different time point

(1)12h：

568.00ug/ml:0.3790,0.2289,0.3042,0.3288,0.0674,0.2201
284.00ug/ml:0.2762,0.2324,0.4608,0.2589,0.2593,0.3209
142.00ug/ml:0.3123,0.3206,0.2267,0.2890,0.3796,0.3392
71.00ug/ml:0.4930,0.3415,0.4555,0.5022,0.4554,0.5655
35.50ug/ml:0.4815,0.5079，0.9200,0.5413,0.4834,0.6310
17.55ug/ml:0.6349,0.5677,0.5458,0.7293,0.7322,0.6952
8.88ug/ml:0.5447,0.7959,0.9297,1.0672,0.9782,0.6132
0.00ug/ml:0.8579,0.8445，0.8377,0.5216,1.2558,0.7909

(1)24h：

568.00ug/ml：0.3247,0.3783,0.3787,0.3776,0.3137,0.4090
284.00ug/ml：0.4623,0.4601,0.4532,0.4472,0.4727,0.4762
142.00ug/ml: 0.4396,0.4662,0.6419,0.7133,0.7463,0.3958
71.00ug/ml: 0.5585,0.6043,0.4870,0.4241,0.7089,1.4660
35.50ug/ml: 0.7289,0.7879,0.6785,0.4888,0.5845,0.7272
17.55ug/ml: 0.8376,0.8763,0.5340,0.8825,0.8783,0.9906
8.88ug/ml:0.6554,0.9758,0.8982,0.8356,1.3987,1.0045
0.00ug/ml: 0.9782,1.1131,0.6869,0.8288,1.2622,1.2583

(1)36h：

568.00ug/ml： 0.4119,0.4286,0.0704,0.2979,0.5447,0.3828
284.00ug/ml： 0.4257,0.3087,0.5311,0.3245,0.4239,0.3604
142.00ug/ml: 0.4517,0.4079,0.4678,0.4011,0.3003,0.5724
71.00ug/ml: 0.4506,0.7691,0.5601,0.5828,0.5789,0.6714
35.50ug/ml: 0.5745,0.8494,0.7979,0.7243,0.5531,0.5665
17.55ug/ml: 0.7819,0.9067,0.8211,0.5360,1.2877,0.5924
8.88ug/ml: 0.6934,0.8828,1.374,0.7188,0.8928,0.8747
0.00ug/ml: 1.796,1.3648,1.3876,1.6806,1.4187,1.0223

Apoptotic rate of A549 cells treated with different concentrations of GJXTL for 24h

GJXTL（ug/mL）	Viable cells（%）	Early apoptotic cells（%）	Advanced apoptotic cells（%）
	94.18	0.41	1.82
0.00	95.78	0.25	1.95
	97.44	0.57	1.81
	87.80	1.31	3.65
142.00	85.59	1.50	4.49
	77.38	1.52	5.78

	90.02	1.81	5.85
284.00	78.05	2.76	8.99
	72.08	3.14	9.09
	69.31	4.01	14.39
568.00	72.86	5.48	12.06
	72.41	6.35	10.33

Effects of different concentrations GJXTL on grey level of STAT3,p-STAT3 and VEGF protein activity in JAK2/STAT3 signal pathway

GJXTL（ug/mL）	β -actin	STAT3	P-STAT3	VEGF
	9.2221	9.2668	7.5701	11.6636
0.00	8.5635	9.1232	6.3524	10.2356
	9.9845	8.5621	5.0526	9.9856
	9.8248	8.9981	6.0411	9.1561
142.00	10.2352	8.2546	6.2531	8.2456
	9.4563	9.0213	5.0213	8.5624
	10.7833	8.2099	7.1135	8.8865
284.00	10.0833	8.7565	4.8963	7.8546
	9.1203	8.6542	5.2132	7.0546
	8.7233	9.3377	4.0306	6.0324
568.00	8.6532	9.0213	4.0213	5.5632
	8.9532	8.5986	3.8546	5.4635

Effects of different concentrations GJXTL on grey level of STAT3,p-STAT3 and VEGF protein activity in JAK2/STAT3 signal pathway

GJXTL（ug/mL）	STAT3	p-STAT3	VEGF	β-actin
	9.2668	7.5701	11.6636	9.2221
0.00	9.1232	6.3524	10.2356	8.5635
	8.5621	5.0526	9.9856	9.9845

	8.9981	6.0411	9.1561	9.8248
142.00	8.2546	6.2531	8.2456	10.2352
	9.0213	5.0213	8.5624	9.4563
	8.2099	7.1135	8.8865	10.7833
284.00	8.7565	4.8963	7.8546	10.0833
	8.6542	5.2132	7.0546	9.1203
	9.3377	4.0306	6.0324	8.7233
568.00	9.0213	4.0213	5.5632	8.6532
	8.5986	3.8546	5.4635	8.9532

Effects of different concentrations GJXTL on relative grey level of STAT3,p-STAT3 and VEGF protein activity in JAK2/STAT3 signal pathway

GJXTL（ug/mL）	STAT3	P-STAT3	VEGF
	1.0048	0.8209	1.2647
0.00	1.0654	0.7418	1.1953
	0.8575	0.6109	1.0001
	0.9159	0.6149	0.8241
142.00	0.8065	0.4856	0.8056
	0.9540	0.5310	0.9055
	0.7614	0.6597	0.8247
284.00	0.8684	0.4856	0.7790
	0.7782	0.4688	0.7735
	1.0704	0.4620	0.6915
568.00	1.0425	0.4647	0.6429
	0.9604	0.4305	0.6102

The tumor volume of different concentrations of GJXTL against A549 cells at different time point (mm^3)

Group	n	D1	D5	D9	D13	D17	D21
	7	115.2	123.72	152.21	204.86	319.23	474.51
Control		82.56	87.35	100.55	228.30	282.08	275.85
		89.04	135.23	189.87	497.62	609.13	760.64

		59.20	95.86	134.56	248.84	340.07	411.60
		67.2	82.56	91.50	243.68	490.92	786.16
		114.22	118.01	121.50	369.98	590.86	821.74
		96.00	120.35	151.29	175.50	301.94	368.37
	9	66.26	70.56	110.86	137.46	152.89	188.32
		99.45	130.25	180.59	192.12	254.78	297.44
		78.03	82.31	96.75	124.00	172.34	202.68
		59.39	75.42	119.48	188.75	214.93	201.11
		167.27	170.25	187.55	445.18	521.46	757.73
DDP		93.15	115.32	151.42	220.47	290.83	359.74
		129.71	145.31	185.00	323.64	390.21	419.90
		121.50	122.45	132.06	253.67	321.86	390.04
		129.65	132.38	137.5	151.23	199.25	208.5
	7	107.8	100.2	105.84	190.68	221.81	272.92
		81.56	78.52	98.88	141.66	220.17	292.67
		71.35	62.31	75.29	98.21	102.35	119.48
GJXLT-low		90.50	110.23	148.72	330.43	512.21	871.79
		54.78	78.93	118.19	131.07	180.23	178.93
		115.2	121.30	127.5	309.66	445.53	753.59
		73.41	110.25	177.5	424.34	475.56	627.20
	8	84.53	108.96	175.11	184.88	285.35	350.75
		56.59	105.63	126.67	140.56	205.46	270.36
		68.69	72.56	128.22	130.79	195.89	260.99
		10.00	15.23	21.87	51.84	96.86	101.88
GJXLT-middle		130.00	132.14	224.49	307.00	386.85	380.7
		135.87	185.2	257.55	335.92	390.65	465.79
		116.31	131.23	210	229.9	262.82	335.73
		27	31.20	80.62	97.49	115.68	193.87
		94.19	156.25	244.80	346.8	407.93	509.06
		21.87	28.56	40.5	49.01	62.84	76.67
		110.26	134.65	167.50	211.14	227.49	247.84
		119.48	135.65	177.5	240.66	285.54	314.47
GJXLT-high	8	110.48	132.85	172.5	268.04	300.08	312.11
		92.60	100.31	105.3	195.96	280.56	301.18
		149.76	156.23	182.07	187.27	259.12	282.96
		18.75	26.53	36.9	97.2	103.02	110.59

The stripped tumor volume of different concentrations of GJXTL against A549 cells (mm^3)

Group	The stripped tumor volume
	204.12
	867.70
	176.72
Control	732.64
	278.3
	309.35
	663.26
	366.77
	173.60
	273.10
	287.18
DDP	179.40
	236.88
	352.11
	296.45
	601.04
	409.73
	172.87
GJXLT-low	106.60
	818.50
	848.38

	244.95
	126.96
	450.17
	323.54
	245.24
GJXLT-middle	365.92
	253.28
	146.45
	77.63
	158.02
	272.41
	206.23
	272.82
GJXLT-high	67.74
	406.08
	283.17
	96.66
	140.05

The positive expression rates of Ki-67（%）

Control	DDP	GJXLT-low	GJXLT-middle	GJXLT-high
72.35	63.52	56.58	58.96	35.56
68.45	68.72	53.26	45.23	30.24
70.68	75.56	65.23	38.98	28.89
69.52	65.35	45.69	40.23	26.53
50.25	53.26	62.35	39.65	28.51

Body weight increase in A549 xenograft mice

Group	n	The body weight before the experiment(g)	The body weight after the experiment(g)	The body weight increase in A549 xenograft mice（%）
		19.5	21.5	10.25
		17.2	18.1	5.23
		18.6	21.7	16.67
Control	7	21.4	22.4	4.67
		14.0	16.4	17.14
		15.7	15.2	3.28
		15.4	17.9	16.23
		17.6	15.4	-12.5
		18.7	15.5	-17.11
		18.1	15.3	-15.47
		14.4	13.4	-6.94
DDP	9	15.2	11.5	-24.34
		13.5	12.1	-10.37
		18.6	14.2	-23.65
		14.0	10.1	-27.85
		15.9	11.3	-28.93
		17.8	20.2	13.48
		18.5	20.6	10.19
		11.3	16.1	42.48
GJXLT-low	7	16.3	19.0	14.21
		14.8	15.8	6.76
		15.6	17.4	11.54
		15.0	17.8	18.67
		18.8	21.5	14.36
GJXLT-middle	8	19.4	21.0	8.25
		15.6	20.0	28.21
		18.1	22.1	22.10

		16.4	17.6	7.31
		14.5	17.3	8.23
		18.1	19.0	4.97
		20.8	22.0	5.77
GJXLT-high	8	18.8	21.5	14.36
		19.4	21.0	8.25
		15.6	20	28.20
		18.1	22.1	22.10
		16.4	22.2	35.37
		14.5	17.6	21.38
		15.7	17.3	10.19
		20.8	19.0	
